# Performance of antiretroviral drugs supply chain management and related challenges in Amhara National Regional State, Ethiopia. In the case of public health facilities found in the Central Gondar zone

**DOI:** 10.1016/j.rcsop.2025.100570

**Published:** 2025-01-21

**Authors:** Meseret Tilahun Zeleke, Berhanemeskel Weldegerima Atsbeha, Belachew Yebeyin Melaku, Yesuneh Tefera Mekasha, Abibo Wondie Mekonen, Shimelis Dagnachew Nigatu

**Affiliations:** a Department of Social and Administrative Pharmacy, School of Pharmacy, College of Medicine and Health Science, University of Gondar, Gondar, Ethiopia; b Department of Logistics and Supply Chain Management, College of Business and Economics, University of Gondar, Gondar, Ethiopia; c Pharmaceutical sciences, Pharmaceutical Analysis and Regulatory Affairs, University of Gondar; P.O.Box=196, Gondar, Ethiopia; d Department of veterinary pharmacy, Pharmaceutical Supply Chain Management, College of Veterinary Medicine and Animal Science, University of Gondar, Gondar, Ethiopia; e Department of veterinary pathobiology, College of Veterinary Medicine and Animal Science, University of Gondar, Gondar, Ethiopia

**Keywords:** Supply chain performance, ARV drugs, Public health facilities, Ethiopia

## Abstract

**Background:**

Effective human immunodeficiency virus (HIV) treatment depends on uninterrupted delivery of prescribed antiretroviral (ARV) drug regimens at anti-retroviral therapy (ART) sites. However, interruptions in access and stock outs are the major challenges in the supply chain of antiretroviral drugs. The issues are very high, particularly in developing countries like Ethiopia. Therefore, this study aimed to assess the supply chain performance of ARV drugs and its challenges in public health facilities in the Central Gondar Zone.

**Methods:**

A descriptive cross-sectional survey complemented by a qualitative research approach was conducted in 30 health facilities (7 hospitals and 23 health centers) that provide ART services. Structured self-administered questionnaires and observational checklists were used to collect quantitative data, while qualitative data were collected using semi-structured open-ended interview guides. Quantitative data was analyzed using SPSS version 26 and Microsoft Excel 2013. A thematic approach was used to examine the qualitative data, which required careful reading of the transcripts to find important themes.

**Results:**

This study revealed that, at the surveyed health facilities, the availability and utilization of bin cards were found to be 100 %. Bin card updating in the surveyed facilities was 90.4 % and 87.2 % on average at hospitals and health centers, respectively. The study found that stock outs of ARV drugs were high in hospitals (16.14 %) as compared to health centers (9.2 %). Lamivudine (3TC) + Dolutegravir (DTG) + Tenofovir (TDF) (300 mg + 50 mg + 300 mg) of 30 tablets was the most frequently stocked-out drug at hospitals and health centers in about 57 % and 39 %, respectively. About 42.8 % of hospitals and 26 % of health centers placed at least two emergency orders within the previous six months at the time of data collection. The storage condition of ARV drugs was not good (68.99 %) in health centers. However, it was better in hospitals and met the criteria for good storage conditions (89.52 %).The qualitative findings also revealed that inadequate infrastructure, a shortage of trained and qualified staff, and a lack of vehicles were the major challenges.

**Conclusion:**

Overall, the study indicated that the surveyed health facilities were not satisfied with the timely updating of bin cards. Additionally, it identified issues such as stock outs of ARV drugs, poor storage conditions (particularly in health centers), inadequate infrastructure, a shortage of trained and qualified staff, and a lack of vehicles. Therefore, to improve the performance of ARV drug supply chain management, all the concerned bodies should work cooperatively in maintaining quality data and be dedicated to updating inventory recording tools, creating standard storage conditions, as well as recruiting pharmacy professionals, facilitating capacity-building training, and offering ongoing mentorship and supervision. Suppliers should maintain adequate inventory at their hub and collaborate with healthcare facilities. The healthcare facilities' should utilize automated inventory-controlling systems.

## 1 Introduction

Life-saving medicines such as ARV drugs are mandatory for allowing people to live with Human Immunodeficiency Virus/Acquired Immune Deficiency Syndrome (AIDS). At the end of 2022, there were an estimated 39.0 million (33.1–45.7 million) people living with HIV, two-thirds of whom (25.6 million) are found in the region of Africa. About 480,000–880,000 people died from HIV-related causes, and 1.3 million (1.0–1.7 million) people acquired HIV.^1^^,^^2^ Therefore, to save the lives of patients, an effective supply chain of ARV drugs and uninterrupted delivery of prescribed ARV drug regimens at anti-retroviral treatment sites should be maintained. Unfortunately, due to improper drug supply management and poor inventory control systems, ART services are mostly facing shortages of ARVs. A number of factors have been described for the discontinuity of ART; among the major factors are the interruption in access and the frequent stock out of ARV drugs.^3^

To ensure an effective supply chain for ART and to protect the wastage of limited public resources requires the involvement of committed staff, international and national stakeholders, and policymakers. Even if holding adequate inventory serves as a buffer against uncertain and fluctuating stocks, the expense associated with supply chain management (SCM), financing, and maintaining inventories is a substantial part of ART services success.^4^

The increasing demand for global AIDS treatment poses extraordinary challenges for ARV drug SCM because each and every service delivery point of ARV drugs (i.e., health centers/clinics, hospitals, and community outreach workers) must have access to a customized and predictable supply of ARVs at all times.^5^ It is obvious that effective pharmaceutical supply management and inventory control avoid stock outs, losses due to unnecessary expiry, and theft and ensure that the desired pharmaceutical products are available at all times in adequate quantity.^6^ Particularly in low- and middle-income countries, supply chain management of ARV becomes increasingly difficult due to an increasing number of ART, an increasing number of ART sites, the diverse nature of the treatment regimen, and a limited capacity.^7^

Poor supply management of ARVs exposes the health of the public to risks, causes the development of ARV drug resistance, hampers progress towards universal access, and diminishes the credibility of ART programmers in the eyes of patients, the community, and healthcare providers. It also puts individual patients at risk of disease progression and death.^1^ Unsatisfactory data records, stock-outs, interrupted reports, inaccurate inventory, and wastage rates were indicators of defective supply chain management of HIV/AIDS commodities. Research conducted in Addis Ababa showed that there was no adequate data on patient medication records and the stock status of HIV/AIDS-related commodities. Moreover, there were frequent stock-outs of ARV medicines and HIV test kits, which indicate the presence of weak SCM.^8^^,^^9^ Another study in Ethiopia shows that HIV test kits and ARV medication are wasted due to expired, lost, or destroyed at health facilities (HFs).^10^

Currently, due to the prevalence of HIV/AIDS, ARV drugs are widely used; in such cases, effective ARV drug supply chain management and inventory control at each service delivery point are very essential. Because solid pharmaceutical supply chain management is a key for uninterrupted access to ARV drugs, avoidance of losses due to expiry and theft, and ensuring the availability of pharmaceutical products in adequate quantity and quality. Up-to-date information about the performance of ARV drug supply chain management in the context of health facilities served as inspiration and was significantly important to addressing related issues. Additionally, the results obtained from such types of studies should be used by policymakers and program managers to realize the implementation of effective supply chain management strategies in minimizing negative outcomes associated with poor pharmaceutical supply chain management. However, in Ethiopia, research related to pharmaceutical supply chain performance, particularly ARV drug supply chain management performance and related factors, is limited. Even to the best of the researcher's knowledge, there was no study conducted in the health facilities in the current study area, Amhara Regional State. Therefore, the aim of this study was to assess the performance of supply chain management of ARV drugs and related challenges in relation to different indicators like order fill rate, lead time, and frequency of emergency order, stock out rate, inventory accuracy rate, and storage conditions of HFs.

## 2 Methodology

### 2.1 Study area and period

The study was conducted in public health hospitals and health centers found in the Central Gondar Zone, Amhara regional state, Ethiopia, from March 15, 2023, to September 30, 2023. The study area is located in the northern part of Ethiopia. Administratively in the study area, there are 13 woredas and one city administration (Gondar city administration). According to the 2023 report acquired from the Amhara regional health bureau and Central Gondar zone health office, in the study area there were a total of 537 health facilities (84 health centers, 423 health posts, 10 hospitals) providing public health care service for over 8 million people. The first health center built in Ethiopia, namely called Kolla Diba Health Center, is found in the current study area. Among the health facilities found in the Central Gondar zone, 9 hospitals and 25 health centers are providing ART programs.

### 2.2 Study design

A mixed-method research approach was carried out to collect the necessary data. The data was collected at public health facilities using a self-administered questionnaire, physical observation using observational checklists, and a face-to-face interview guide with key informants (KIs). The study mostly used a quantitative approach to produce numerical data with regard to ARV drugs supply chain performance indicators such as inventory management and/or recording tools and storage conditions, while a qualitative approach was used to explore the challenges faced in the study area and strengthen the quantitative result.

### 2.3 Source and study population

#### 2.3.1 Source population

All public health facilities providing ART services in the study area, all pharmacy professionals who were working in the health facilities, all documents used for stock record keeping of ARV medicine, and all ARV medicines in the facilities were source populations.

#### 2.3.2 Study population

Public health facilities providing ART services, pharmacy professionals working in ART pharmacies, first- and second-line ARV drugs, bin card records, and report and requisition forms (RRFs) were the selected health facilities that were the study subjects.

### 2.4 Eligibility criteria

#### 2.4.1 Inclusion criteria

Public health facilities that provide ARV treatment and pharmacy personnel working in ART pharmacies more than 6 months prior to data collection time were included. First- and second-line ARV medicines, bin card records, and RRFs available from the last 6 months prior to data collection time at the selected health facilities were included in the study.

#### 2.4.2 Exclusion criteria

Health posts were excluded because they are used as dispensing units of health centers, and their inventory management practices could not be consistent, unlike hospitals and health centers. Healthcare facilities that were experiencing political instability and safety problems were excluded.

### 2.5 Sample size and sampling technique

#### 2.5.1 Sample size determination

Regarding health facilities, the guide for conducting assessments of supply chain performance using the Logistic Indicator Assessment Tool (LIAT) was considered. According to the principle of LIAT, to get a representative sample of the population, at least 15 % of the target facilities should be included in the study sample, and if the study is conducted at the country level, a minimum of 100 health facilities should be included. However, in the case of this study, it is difficult to apply the concept of LIAT since there are 34 health facilities delivering ART services, and some of those are excluded based on the eligibility criteria. As the target populations are limited in number, purposefully, 30 health facilities that fulfilled the inclusion criteria were included in the study population to collect the necessary data.

Participants in the study were purposefully chosen based on their direct involvement in pharmaceutical supply chain management activities and their position in the facilities. Accordingly, the heads of pharmacies, ART store managers or general store managers, and ART dispensers from the selected hospitals and health centers participated to collect the primary data. To collect the secondary data, the recommendation of LIAT (i.e., a minimum of six-month logistic reports should be considered for service assessment of health facilities) and according to Ethiopia's integrated pharmaceutical logistics system (IPLS) (i.e., each hospital and health center is expected to submit one RRF report every two months to the pharmaceutical supply agency's (EPSA) hub). Therefore, each health facility is required to submit three RRF reports in six months (i.e., 1 RRF report every two months for six months). Accordingly, a total of 90 RRF reports were submitted to EPSA hubs at 30 health facilities (3 × 30 = 90). In addition, 450 bin card records (15 bin card records of 15 ARV drugs available from 30 health facilities) were assessed to collect the data (Fig. 3). For qualitative data collection, the number of participants was determined based on the viewpoints of different researchers and criteria required for qualitative reports. In qualitative inquiries, the number of interviewed participants is suggested to be 10 to 30 people.^11^, ^12^, ^13^ The study therefore came up with the sample of 12 KIs because of the saturation of information.

#### 2.5.2 Sampling technique

Due to the limited number of target populations that fulfill the inclusion criteria, the health facilities were included through a census rather than sampling from the population. The purposeful sampling technique was utilized to select participants based on their direct involvement in ART supply chain management activities and their position in the facilities. Regarding KIs, pharmacy heads and store managers were involved in the in-depth interview to generate qualitative data. The selection is because they are rich in information and assigned to the position of a decision-maker.

### 2.6 Data collection tools and procedures

#### 2.6.1 Data collection tools

The standard criteria from the LIAT developed by the USAID | Deliver Project and data collection tools from various related articles and research were also referred to in the adoption of the data collection tool^1^, 2., 3^,^^14^^,^^15^. Structured self-administered questionnaires, observational checklists, and semi-structured open-ended interview guides were used to collect the required data.

#### 2.6.2 Data collection procedures

Two pharmacists and the principal investigators were allocated and participated as data collectors for quantitative and qualitative data, respectively. The consent form was prepared, and communication was done with respondents to get their consent. Once participant consent was obtained, the prepared questionnaires were distributed to each participant, and then the quantitative data, like logistic recording updating practice, frequency of emergency order, stock out rate, and storage condition, were collected using structured questionnaires and observational checklists. The ART dispenser, pharmacy store manager, and head of the pharmacy from the sampled facilities participated in filling out the questionnaire, whereas an in-depth interview with flexible probing techniques was designed to collect the qualitative data from the key informants. Each interview, on average, lasted for 20 min. The interviews were conducted by the principal investigator to maintain consistency. The required data was collected through text notes and audio recordings.

### 2.7 Data validity and quality assurance

The study questionnaire and interview guide were derived from a standard tool and developed after reviewing previously studied related research.^1^^,^^3^^,^^8^^,^^14^^,^^16^To maintain the quality of the data and to encourage meaningful participation by the respondents, the layout of the questionnaires was kept clear and very simple. The principal investigator provided two hours of training to data collectors on data collection procedures and the significance of the study. Prior to data collection, a pretest was carried out on 5 % of the study facilities, which were not included in the actual data collection. During data collection time, the misunderstood questions have been clarified in detail every day during the data collection. The collected data was carefully checked, cleaned for completeness, summarized, labeled, and coded before being entered into Statistical Packages for Social Science (SPSS) and MS Excel. To ensure the trustworthiness of qualitative data, the probing and flexible questions and interview guide were prepared in a clear way, and it was also interpreted into the local working language (Amharic). Additionally, the collected data was maintained for its quality through proper document processing, carefully checked for completeness and consistency, and detailed audit trails were also made. For better understanding of the perception of KIs and to clearly report their insight, the principal investigator (qualitative data collector) read and followed qualitative data reporting guidelines.^13^

### 2.8 Data processing and analysis

Standard indicators (logistic recording updating practice, frequency of emergency order, lead time, order fill rate, a stock out rate, and storage conditions) that are used in this study to measure the supply chain performance of ARV drugs at the facility level are quantifiable based on the principle of LIAT.^11^, ^12^, ^13^ The quantitative data was recorded into an Excel spreadsheet, and the parameters were analyzed using SPSS version 26. Descriptive statistics such as the frequency and percentage obtained from respondents and secondary data (obtained from review of facility documents) were computed as per the standard operating procedure of the integrated pharmaceutical logistics system of Ethiopia as described in the equation below. Tables, graphs, and charts were used to summarize the demographic information of respondents and the facility profile.

$$\text{Logistic recording updating practice}=\frac{\mathrm{Total number of recored tool updated}\;}{\mathrm{Total numbr of recorded tool used}\;}x100$$

$$\text{Frequency of emergency order}=\frac{\mathrm{Number of health facilities faced to emergency order}\;}{\mathrm{total number of facilities}\;}x100$$

$$\text{Stock out rate}=\frac{\mathrm{Number of}\;\mathrm{HFs}\;\mathrm{that experianced stock out for}\;\mathrm{ARV}\;\mathrm{product}}{\mathrm{Total number of}\;\mathrm{HFs}\;\mathrm{ecpected to need the product}}x100$$

$$\text{Storage condition}=\frac{\mathrm{Number of faccilities fulfiiled acceptable storage condition criiteria}}{\mathrm{Number of facilities surveyed}\;}x100$$

$$\text{order fill rate}=\frac{\mathrm{Number of orders filled correctly}}{\mathrm{Total number of order placed}\;}x100$$

For qualitative data, prior to identifying the themes, the data collected by text notes were repeatedly read, and the audio records were listened to several times and transcribed from the voice recorder. Then the data obtained from different KIs were organized thematically. Accordingly, based on its characteristics and the perception of KIs, the qualitative data were organized into three major thematic areas, including challenges related to ARV drug supply, infrastructure and administration, and personnel-related challenges.

## 3 Results

### 3.1 Socio-demographic profile of facilities and participants

For this study, a total of 30 health facilities, 23 (76.66 %) health centers and 7 (23.33 %) hospitals, were assessed. Regarding study participants, a total of 30 pharmacy professionals were involved. Of these, the majority, 15 (50 %) of the participants, were ARV drug dispensers, and the remaining 8 (26.7 %) and 7 (23.3 %) were pharmacy heads and store managers, respectively. Concerning their level of education, more than half (16 (53.3 %)) were at the diploma level, and the majority (11 (36.7 %)) of the respondents have 1–5 years' work experience in relation to ARV drugs (Table 1).

**Table 1:** Socio - demographic profile of health facilities and participants.

Variable	Category	Frequency (n) and percentage (%)
Health facility	Health center	23 (76.66 %
	Hospital	7 (23.33 %
Gender	Male	16 (53.3 %)
	Female	14 (46.7 %)
Level of education	Diploma	16 (53.3 %)
	Bachelor degree in Pharmacy	13 (43.3 %)
	Master of science	1 (3.3 %)
Work experience	< 1 year	2 (6.7 %)
	1–5 years	11 (36.7 %)
	5–10 years	10 (33.3 %)
	>10 years	7 (23.33 %)
Position of respondents	Drug dispenser	15 (50.0 %)
	Drug store personnel	7 (23.3 %)
	Head of the pharmacy	8 (26.7 %)
IPLS training	Trained	20 (66.6 %)
	Not trained	10 (33.3 %)

Note: B.Pharm: Bachelor of pharmacy, Msc: Master of Science, IPLS: Integrated pharmaceutical logistic system.

### 3.2 Assessment of inventory management tools of ARV drugs

There are different types of standard recording and reporting formats to be used for the proper functioning of pharmaceutical supply chain management. The availability and proper utilization of standard formats like bin cards, RRFs, and internal facility report and resupply forms (IFRR) are good indicators of supply chain performance.

#### 3.2.1 Availability, utilization and updating of bin cards

This study revealed that the availability of bin cards was found to be very high, 100 %, both at hospitals and health centers. All hospitals and health centers reviewed utilized inventory recording bin cards at the time of visit. The average bin card used for ARV medicine at both hospitals and health centers was 100 %. The percentage of facilities with bin cards updated was 90.4 % and 87.2 % in hospitals and health centers, respectively. However, in all the surveyed health facilities, the practices of bin card updating were done manually, with no facilities using electronic systems to update bin cards. The bin card was updated to Lamivudine (3TC) + Dolutegravir (DTG) + Tenofovir (TDF) (300 mg + 50 mg + 300 mg) of 30 tablets and Lamivudine (3TC) + Dolutegravir (DTG) + Tenofovir (TDF) (300 mg + 50 mg + 300 mg) of 90 was 100 % both at the hospital and health center (Table 2).

**Table 2:** Availability and updating of bin card by facility and product type.

Product type	Availability of bin card at hospital and health center (%)	Bin card updating at hospital n (%)	Bin card updating at health center n (%)
Abacavir - 300 mg – Tablet	100 %	4 (80 %)	17/19 (89.4 %)
Abacavir (ABC) + Lamivudine (3TC) - (120 mg + 60 mg) - Tablet (Dispersible)	100 %	7 (100 %)	17/19 (89.4 %)
Atazanavir(ATV) + Ritonavir (RTV) (300MG + 100 mg)	100 %	6 (85.7 %)	16/19 (84.2 %)
Dolutegravir (DTG) - 50 mg – Tablet	100 %	7 (100 %)	16/19 (84.2 %)
Lamivudine + Efavirenz +Tenofovir `(300 mg + 400 mg + 300 mg) – Tablet	100 %	6 (85.7 %)	16/19 (84.2 %)
Lamivudine (3TC) + Dolutegravir (DTG) + Tenofovir (TDF) - (300 mg +50 mg + 300 mg) of 30 tablet – Tablet	100 %	7 (100 %)	23/23 (100 %)
Lamivudine 150 mg tablet	100 %	6 (85.7 %)	16/19 (84.2 %)
Lamivudine (3TC) + Dolutegravir (DTG) + Tenofovir (TDF) - (300 mg +50 mg + 300 mg) of 90	100 %	7 (100 %)	23/23 (100 %)
Lamivudine + Tenofovir - (300 mg + 300 mg) – Tablet	100 %	7 (100 %)	16/19 (84.2 %)
Lamivudine + Zidovudine - (150 mg + 300 mg) – Tablet	100 %	6 (85.7 %)	20/20 (100 %)
Lamivudine + Zidovudine - (30 mg + 60 mg) – Tablet	100 %	6 (85.7 %)	19/19 (100 %)
Lopinavir + Ritonavir - (100 mg + 25 mg)	100 %	6 (85.7 %)	15/19 (78.9 %)
Lopinavir + Ritonavir - (200 mg + 50 mg)	100 %	6 (85.7 %)	16/19 (84.2 %)
Lopinavir + Ritonavir - (40 mg + 10 mg)	100 %	6 (85.7 %)	15/19 (78.9 %)
Ritonavir (RTV) - 100 mg - Tablet	100 %	6 (85.7 %)	15/19 (78.9 %)
Average	100 %	90.4 %	88 %

#### 3.2.2 Availability and utilization of reporting formats

This study assessed the availability and utilization of reporting and ordering formats like RRF and IFRR. The central warehouse's hub with the health facilities and the health facilities' with their dispensing unit can communicate with each other about the status and stock level of ARV drugs in RRF and IFRR formats, respectively. The study found that the entire reporting format was available in all surveyed health facilities. RRF and IFRR were used and completed 100 % in hospitals, and the percentage of using the RRF format regularly in health centers is also shown at 100 %. However, IRRF utilization at the health center was about 96.7 %.

#### 3.2.3 Accuracy of data record on bin cards

The quality of the data recording on the bin card was assessed by cross-checking the accuracy of the bin card balance with the physical inventory count for each of the 15 selected ARV drugs. As mentioned in Table 3, below, the percentage of bin card updates at hospitals and health centers was 90.4 % and 88 %, respectively. The high average percentage of discrepancy in ARV medicine in hospitals (15.8 %) as compared to health centers (11.3 %) was observed when the balance on the updated bin card was cross-checked with the physical inventory value observed on the day of visit. Among ARV medicines involved in this study, a high percentage of discrepancy was observed in lamivudine 150 mg tablets and lopinavir + ritonavir (200 mg + 50 mg) (16.6 %) at the hospital, and a low percentage of discrepancy was observed in lamivudine + tenofovir (300 mg + 300 mg) tablets and lopinavir + ritonavir (100 mg + 25 mg) was 10.5 % at the health center (Table 3).

**Table 3:** Percentage of health facilities with updated bin cards and discrepancy in the data record by product and facility type, Gondar, 2023.

Product type	Update of bin card at Hospitals n (%)	Hospitals with discrepancy n (%)	Update of bin card at HC	HC with discrepancy (n)%
Abacavir - 300 mg – Tablet	6 (85.7 %)	0	17/19 (89.4 %)	0
Abacavir (ABC) + Lamivudine (3TC) - (120 mg + 60 mg) - Tablet (Dispersible)	7 (100 %)	0	17/19 (89.4 %)	0
Atazanavir(ATV) + Ritonavir (RTV) (300MG + 100 mg)	6 (85.7 %)	0	16/19 (84.2 %)	0
Dolutegravir (DTG) - 50 mg – Tablet	7 (100 %)	0	16/19 (84.2 %)	0
Lamivudine + Efavirenz +Tenofovir `(300 mg + 400 mg + 300 mg) – Tablet	6 (85.7 %)	0	16/19 (84.2 %)	0
Lamivudine (3TC) + Dolutegravir (DTG) + Tenofovir (TDF) - (300 mg +50 mg + 300 mg) of 30 tablet – Tablet	7 (100 %)	0	23/23 (100 %)	3/23(13 %)
Lamivudine 150 mg tablet	6 (85.7 %)	1/7 (14.2 %)	16/19 (84.2 %)	0
Lamivudine (3TC) + Dolutegravir (DTG) + Tenofovir (TDF) - (300 mg +50 mg + 300 mg) of 90	7 (100 %)	0	23/23 (100 %)	0
Lamivudine + Tenofovir - (300 mg + 300 mg) – Tablet	7 (100 %)	1/7 (14.2 %)	16/19 (84.2 %)	2/19 (10.5 %)
Lamivudine + Zidovudine - (150 mg + 300 mg) – Tablet	6 (85.7 %)	0	20/20 (100 %)	0
Lamivudine + Zidovudine - (30 mg + 60 mg) – Tablet	6 (85.7 %)	0	19/19 (100 %)	0
Lopinavir + Ritonavir - (100 mg + 25 mg)	6 (85.7 %)	0	15/19 (78.9 %)	2/19 (10.5 %)
Lopinavir + Ritonavir - (200 mg + 50 mg)	6 (85.7 %)	1/6 (16.6 %)	16/19 (84.2 %)	0
Lopinavir + Ritonavir - (40 mg + 10 mg)	6 (85.7 %)	0	15/19 (78.9 %)	0
Ritonavir (RTV) - 100 mg – Tablet	6 (85.7 %)	0	15/19 (78.9 %)	0
Average	90.4 %	15.8 %	88 %	11.3 %

Note: HC: Health center.

## 4 Evaluation of supply chain management indicators of ARV drugs

### 4.1 Order lead time

**Fig. 1:**
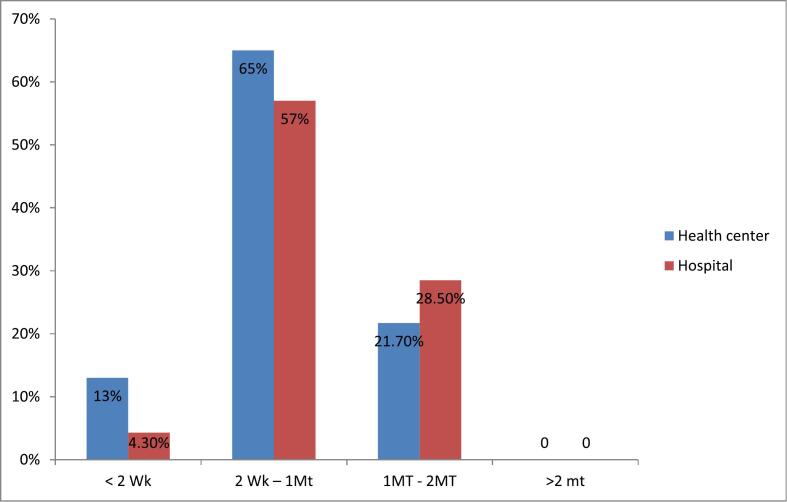
Percentage of facilities with perceived order lead time for ARV drugs by facility type. Note: WK: weak, MT: month.

The current study found that 65 % of health centers and 57 % of hospitals replied that they usually received products requested within two weeks to one month. Some health facilities, 4.3 % of the hospitals, and 13 % of health centers receive their supply within less than 2 weeks. Limited number of health facilities 21.7 % of the health centers and 28.5 % of hospitals reported having to wait more than 1 month to 2 months to receive products after placing orders. The study also revealed that there were no facilities that waited more than 2 months for order resupply (Fig. 1).

### 4.2 Order fill rate

The study revealed that the average percentage of facilities refilled based on their request was 82.4 % and 80.13 % at hospitals and health centers, respectively. The facility order fill rate for Lamivudine, efavirenz, and tenofovir (300 mg + 400 mg + 300 mg) were 80 % at hospitals and 78 % at health centers. The lowest percentage (40 % of refilled) was shown for Abacavir (300 Mg) at the health center and 59 % for Lamivudine (3TC), Dolutegravir (DTG), and Tenofovir (TDF) (300 mg + 50 mg + 300 mg) of 30 tablets at the hospital (Table 4).

**Table 4:** Percentage of facilities with perceived order fill rate for ARV medicine by facility type, Gondar, 2023.

Product name	Percentage of order fill rate of products at HCs (*n* = 23)	Percentage of order fill rate of products at Hospitals (n = 7)
Abacavir - 300 mg – Tablet	40 %	84 %
Abacavir (ABC) + Lamivudine (3TC) - (120 mg + 60 mg) - Tablet (Dispersible)	94 %	86 %
Atazanavir(ATV) + Ritonavir (RTV) (300MG + 100 mg)	44 %	70 %
Dolutegravir (DTG) - 50 mg – Tablet	73 %	84 %
Lamivudine + Efavirenz +Tenofovir - (300 mg + 400 mg + 300 mg) – Tablet	80 %	78 %
Lamivudine (3TC) + Dolutegravir (DTG) + Tenofovir (TDF) - (300 mg +50 mg + 300 mg) of 30 tablet – Tablet	73 %	59 %
Lamivudine 150 mg tablet	100 %	66 %
Lamivudine (3TC) + Dolutegravir (DTG) + Tenofovir (TDF) - (300 mg +50 mg + 300 mg) of 90	66 %	100 %
Lamivudine + Tenofovir - (300 mg + 300 mg) – Tablet	100 %	72 %
Lamivudine + Zidovudine - (150 mg + 300 mg) – Tablet	84 %	82 %
Lamivudine + Zidovudine - (30 mg + 60 mg) – Tablet	100 %	86 %
Lopinavir + Ritonavir - (100 mg + 25 mg)	90 %	100 %
Lopinavir + Ritonavir - (200 mg + 50 mg)	95 %	100 %
Lopinavir + Ritonavir - (40 mg + 10 mg)	87 %	89 %
Ritonavir (RTV) - 100 mg – Tablet	76 %	80 %
Average order fill rate	80.13 %	82.4 %

Note: HCs: Health Centers.

### 4.3 Emergency order

The status of emergency orders in health facilities during the last six months, starting from the time of data collection, was assessed, and the study found that 26 % of health centers and 42.8 % of hospitals placed at least two emergency orders in the previous six months prior to the data collection time, and also 14.2 % of the hospitals placed three emergency orders within the last six months (Table 5).

**Table 5:** Percentage of facilities with perceived emergency order in the last six month by facility type, Gondar, 2023.

Number of EO in the last six month	Health centers (n = 23)		Hospitals (n = 7)	
	Frequency (n)	Percentage (%)	Frequency (n)	Percent (%)
No EO	11	47.8 %	1	14.2 %
One EO	6	26 %	2	28.6 %
Two EO	6	26 %	3	42.8 %
Three EO	0	0	1	14.2 %

Note: EO: Emergency Order

### 4.4 Stock out rate

This study revealed that the average stock out of ARV medicines was 9.2 % and 16.14 % in health centers and hospitals, respectively (Fig. 3). Lamivudine (3TC) + Dolutegravir (DTG) + Tenofovir (TDF) (300 mg + 50 mg + 300 mg) of 30 tablets were the most frequently stocked-out drugs (57 % and 39 %) at hospitals and health centers, respectively (Table 6).

**Table 6:** Percentage of stock out rate of ARV medicine by facility type.

Product name	Percentage of stock out of ARV drug in HC (n = 23)	Percentage of stock out of ARV in Hospitals (*n* = 7)
Abacavir - 300 mg – Tablet	2 (8.6 %)	1 (14.2 %)
Abacavir (ABC) + Lamivudine (3TC) - (120 mg + 60 mg) - Tablet (Dispersible)	1 (4.3 %)	1 (14.2 %)
Dolutegravir (DTG) - 50 mg – Tablet	1 (4.3 %)	3 (42.8 %)
Lamivudine + Efavirenz +Tenofovir - (300 mg + 400 mg + 300 mg) – Tablet	3 (13 %)	3 (42.8 %)
Lamivudine (3TC) + Dolutegravir (DTG) + Tenofovir (TDF) - (300 mg +50 mg + 300 mg) of 30 tablet – Tablet	9 (39.13 %)	4 (47 %)
Lamivudine + Tenofovir - (300 mg + 300 mg) – Tablet	2 (8.6 %)	1 (14.2 %)
Lamivudine + Zidovudine - (150 mg + 300 mg) – Tablet	4 (17.3 %)	2 (28.5 %)
Lamivudine + Zidovudine - (30 mg + 60 mg) – Tablet	7 (30.3 %)	1 (14.2 %)
Lopinavir + Ritonavir - (40 mg + 10 mg)	1 (4.3 %)	1 (14.2 %)
Ritonavir (RTV) - 100 mg – Tablet	2 (8.6 %)	0
Average	9.2 %	16.14 %

Note: ARV: Antiretroviral, HC: Health center

## 5 Assessment of storage conditions of ARV drugs

The storage condition of the HFs was assessed based on visual inspection using 15 standard criteria that are used as good pharmaceutical practices in ARV medicine storage areas. This study indicated that the availability of sufficient space for storage of existing products is only about 6 (26.1 %) in health centers and somewhat high in hospitals (5 (71.4 %) as compared to health centers). The availability of separate stores for active drugs from unwanted and expired items is available only in 12 (52.2 %) and 3 (42.9 %) health centers and hospitals, respectively. From the surveyed facilities, 16 (69.6 %) of health centers and 2 (28.6 %) of hospitals are not visually free from harmful insects and rodents. Moreover, this study revealed that the average storage conditions at hospitals and health centers were 89.52 % and 68.99 %, respectively (supplementary table 1).

**Fig. 2:**
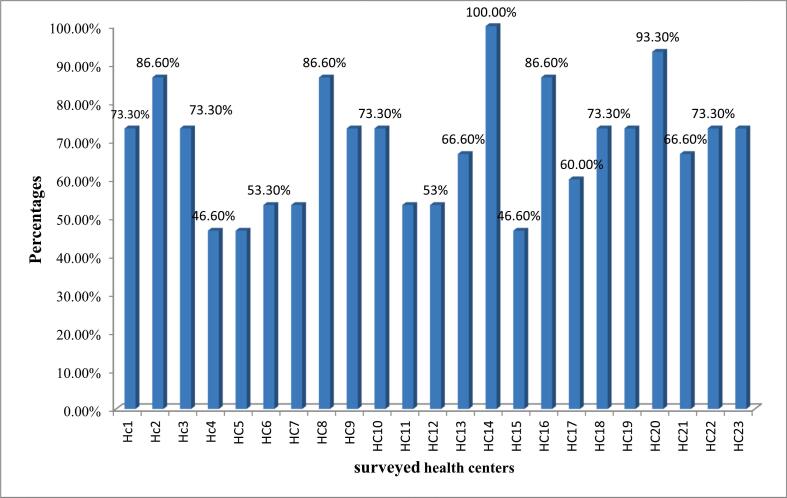
Average storage performance at the surveyed health centers. Note: HC; Health Center

**Fig. 3:**
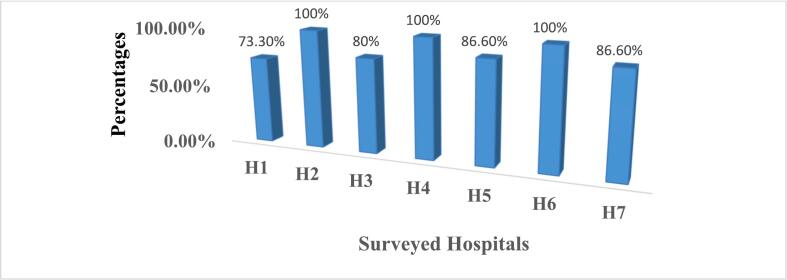
Average storage performance at the surveyed hospitals. Note: H: hospital.

Of the 23 health centers surveyed in this study, only about 5 (21.73 %) of them met the criteria for good storage conditions, and about 3 of these had a storage performance of less than 50 % (Fig. 2). While from 7 hospitals surveyed in this study, only 1 hospital did not meet the acceptable criteria for good storage conditions (Fig. 3).

## 6 Qualitative study result

### 6.1 Assessment of Challenges Faced in the Supply Chain Management Performance of Antiretroviral Drugs

A total of twelve (*n* = 12) KIs were involved in collecting the qualitative data through face-to-face interviews with the aim of identifying and exploring the challenges to the supply chain performance of ARV drugs. The interview was interactive and conducted by the principal investigator using a semi-structured interview. From the KIs, of whom seven were head of the pharmacy and five were store managers. Concerning their level of education, half were in diploma and half at degree level, and the majority (*n* = 11) of the respondents had greater than five years of work experience in relation to ARV drugs. Based on the perception of KIs and characteristics of the collected data the challenges affecting the performance of ARV drugs were divided into three major thematic areas. These were ARV drugs supply, infrastructure and administrative, and personnel related challenges.

### 6.2 Theme one: ARV supply related challenges

Minimum and maximum months of stock have already been established by EPSA; based on this, both hospitals and health centers should have a minimum of 2 months and a maximum of 4 months of stock on hand. This study indicated that even if availability and access to ARV drugs are good, the accessibility of ARV drugs in type was not satisfied across the surveyed health facilities.

### 6.3 The majority of KIs mentioned that

*“When compared to other pharmaceutical products, the availability and accessibility of ARV drugs in our health facility is good. As you are aware, maintaining an uninterrupted supply of ARV medications at ART sites is mandatory because ARV treatment regimens are continuous throughout a patient's lifetime. However, in rare cases, there are limitations in the access of some ARV medications, which makes the provision of successful ART challenging.” (Informants #1, #2, #4, #6, #9, #11, #12).*

Another one KI mentioned that:

*“We occasionally experience inconsistent and interrupted supply, which leads to stocking out of some ARV drugs. This may be due to a short expiration date, inaccurate quantification, and poor supply systems at central EPSA and its hubs. But, as you can understand, being interrupted does not mean a complete cessation of treatment at the facilities. To solve such types of problems, we borrow ARV medicines from another health facility that has enough stock, but such cases increase staff workloads. Sometimes we are forced to give no more than two weeks or a daily dose per patient. In such cases, some patients, particularly those coming from very far/remote areas, become dissatisfied and shout on as.”(Informant #4).*

### 6.4 Theme two: Infrastructure and administrative-related challenges

As reflected by the majority of the KIs, the lack of adequate storage space and vehicle- and administrative-related problems were the challenges faced in the management of ARV medicine in almost all facilities.

Almost three-fourths of KIs mentioned that:

*“We don't have a separate storage area for expired and damaged ARV medicines. There are no adequate and well-furnished shelves for the storage of available ARV drugs. In such a way, it is difficult to maintain the quality of ARV medicines. Always we asked this issue for higher administrative bodies and offices assigned at facilities, zonal, and regional levels, but still we did not get a solution.” (Informant #3, #4, #8, #9, and #12).*

Most KI loudly reflected and emphasized that:

*“The ARV drug storage area is our major problem in our health facility. As you see, our storeroom is not clean; it is not sufficient. We have no separate area for dispensing ARV drugs from other pharmaceuticals; due to this, we are obligated to serve our customers in the same room. This may cause poor patient adherence because of social stigma. Therefore, how can we improve the SCM performance?”* (Informants #1, #2, #3, #6, #7, and #11).

*“Additionally, ARV drug transportation and vehicle-related problems were mentioned as challenges in the ARV drug supply chain. Most KIs reflected that, “EPSA is responsible for transporting ARV medications and related supplies by using their own vehicles. But sometimes, if an emergency order is placed, the supplier/EPSA is not willing to deliver products by their vehicle. As you know, drug transportation vehicles are very essential for healthcare facilities. But as a sector currently, we have no vehicles assigned for this to our facilities. Due to this, we are obligated to use another vehicle and use other alternatives, like vehicle rental or requesting support from other sectors like the agriculture office, civil services office, etc. In such a case, there is no on-time delivery, and it leads to unexpected stock outs of ARV drugs.”*(Informants #4, #5, #6, #7, #8).

### 6.5 Theme 3: Personnel related challenge

The lack of sufficient pharmacy professionals and less commitment of available staff were mentioned as the problems of improving the performance of ARV supply chain management. The shortage of staff, staff workload, lack of training, and lack of educational opportunity were discussed under this theme, and KIs noted the lack of sufficient pharmacy professionals was the problem in most facilities.

Man power related problem:

More than half of KIs mentioned that:

“*A shortage of staff is our major challenge; regarding pharmacy staff in our facility, we do not have sufficient staff as a whole. Due to this, only one pharmacist is engaged in the ART dispensing section, which is enforced to perform both dispensing and recording activities. This creates a workload, and most pharmacists are exhausted and less motivated, so it is difficult to perform daily activities like filling out the daily registration book, updating the bin card, and filling out the RRF on time.” (Informant #1, #2, #4, #6, #9, #11, #12).*

One KI from one health facility emphasized that:

*“In our health center, there are only three pharmacists, but in principle, to perform pharmaceutical-related activities for the health center, at least five pharmacists are mandatory. As you see today, the flow of patients to our health center is very high, which makes it difficult to manage the dispensing, recording, and documentation of both ARV and other pharmaceutical products with these three pharmacists. We frequently asked about staff fulfillment, but still we didn't get a response due to budget constraints.”* (Informant #8).

Almost all KIs emphasized that:

*“On-the-job training, capacity building, and educational opportunities are our problems. It is difficult to provide quality services with previous knowledge obtained from diploma or degree courses. Therefore, training and capacity-building programs and empowering the staff through providing educational opportunities are very essential to motivating the staff and enhancing the quality of services. Due to the lack of such types of opportunities, currently staff turnover and staff skill are our major challenges.”* (Informant # 1, #2,#4, #6,#7, #8, #9,#11,#12).

One KI loudly said that

*“Two years ago, my level of qualification was a bachelor's degree in pharmacy, but currently I have a master of science in pharmaceutical supply chain management. The health center is not willing to sponsor me. Due to this, I sacrificed different things to learn my master's program. I devoted both my time and money. But still, the facilities are not willing to do a top-up for my salary. Currently, my salary is paid based on my previous qualification. To tell the truth, now I will be obligated to leave this health center if I get another opportunity.”(*Informant #3).

Another KI mentioned that

*“I have served as a pharmacist for more than 7 years, but I did not have a training opportunity to improve my professional skills and educational level. Non-governmental organizations (NGOs) and regional health bureaus provided some training opportunities, but these were exclusively available to nurses and medical doctors. Let me tell you one practical example: when abacavir was replaced with lopinavir, only nurses and physicians were selected for that training, but to me, this training was also relevant for pharmacists because we have direct contact with HIV patients to give advice about the use and handling of ARV medicine.”(Informant #5).*

## 7 Discussion and future insights

This study aimed to assess the performance of supply chain and inventory management practices for ARV medicines in public health facilities. Using a bin card is a time-consuming and laborious task; however, implementing this inventory management tool makes the pharmaceutical services more accurate and effective.^16^ This study indicated that both the availability and utilization of bin cards were found to be 100 % at hospitals and health centers, which is in line with the expected target value (100 %). This was similar to the study conducted in East Shewa and Addis Ababa, which indicated that the availability of bin cards was 100 %.^17^^,^^18^ However, in terms of utilization of bin cards, the finding of this study was higher as compared to the results of a study conducted on inventory management of laboratory commodities in Gambela regional state and Jimma zone, utilization of bin cards was 58.8 % and 69.9 %, respectively.^19^^,^^20^ This observed difference could be due to the differences in the commitment of professionals to utilize the available bin cards and the study settings, as the previous study focused only on laboratory commodities.

Regularly updating inventory recording tools is used to know the status of available stock and is good for making evidence-based decisions across health facilities.^21^ The current study revealed that the percentage of updated bin cards was 90.4 % and 87.2 % at hospitals and health centers, respectively. The result is somewhat similar to the result of the study conducted on supply chain management of anti-TB drugs in public health facilities in Addis Ababa, which found that the percentage of updated bin cards was 90.8 % at the hospitals and 82.8 % at the health centers. However, this result is higher as compared to the IPLS national survey, which found that the updating bin card was 73 % at hospitals and 64 % at health centers. The result is also higher than the result of the other study conducted in Jimma, which found that the average bin card updating practices were 38 (55.1 %).^18^^,^^22^^,^^23^ This observed difference might be due to the lack of staff workload and staff turnover at facilities. The low performance in achieving the expected target (100 %) for bin card updating at the currently surveyed health facilities could be due to lack of staff commitment and staff workload.

Majority of KIs highlighted that, “*A shortage of staff is our major challenge; regarding pharmacy staff in our facility, we do not have sufficient staff as a whole. Due to this, only one pharmacist is engaged in the ART dispensing section, which is enforced to perform both dispensing and recording activities. This creates a workload, and most pharmacists are exhausted and less motivated, so it is difficult to perform daily activities like filling out the daily registration book, updating the bin card, and filling out the RRF on time*.”(Informants #1, #2, #4, #6, #9, #11, #12). “As you see today, the flow of patients to our health center is very high, which makes it difficult to manage the dispensing, recording, and documentation of both ARV and other pharmaceutical products with these three pharmacists. We frequently asked about staff fulfillment, but still we didn't get a response due to budget constraints.” (Informant #8).

Data is accurate when there is no discrepancy between stock balances on the bin card record compared with the physical count. The current study revealed that the accuracy rate is 84.2 % and 88.7 % in hospitals and health centers. The result is somewhat similar to the result of the study conducted in Addis Ababa to evaluate HIV rapid test kits inventory management practice and had an average accuracy rate of approximately 84 % on its bin cards.^9^ The discrepancy found in the current study could be due to the practice manual inventory controlling practices, the lack of commitment of staff, and the lack of commitment of staff. Because during our observation in the selected facilities, we noted that the bin card updating practice was manual in all facilities, and KIs also mentioned that “*Due to the limited number of pharmacy professionals, a single pharmacist is engaged in the ART dispensing section to perform both dispensing and recording activities. This creates a workload, and most pharmacists are exhausted and less motivated, so it is difficult to perform daily activities like filling out the daily registration book, updating the bin card, and filling out the RRF.”* (Informants #8 and #4).^3^

If lead time is not managed and planned properly, there will be the existence of pharmaceutical stock outs, unexpected costs, and a reduction in customer satisfaction.^24^The current study found that the majority, 65 % of health centers and 57 % of hospitals, received the products they requested within two weeks to one month. This is comparable to the study conducted in Kenya, which indicated the order lead time ranged from the shortest of 11.3 days to the longest of 23.7 days in the hospital.^25^^,^^26^ But the result is opposite to another study that indicated only 4 % of the facilities reported waiting for more than two months to receive products after placing orders.^27^^,^^28^

One of the key performance indicators used to show the efficiency of the supply chain is the enhanced order fill rate. The current study revealed that the average percentage of facilities refilled based on their request was 82.4 % and 80.13 % at hospitals and health centers, respectively. This is comparable to the study conducted in Addis Ababa, which showed the percentage of refilling according to their request was 80 % at hospitals and 87.7 % at health centers.^8^ However, it was low as compared to the theoretical or ideal (100 %). The most important output of the health supply chain is uninterrupted product access since stock outs of essential medicines lead to treatment interruption, drug resistance, and a reduction in customer satisfaction.^29^ The study revealed that the average stock out of ARV drugs is 9.2 % and 16.14 % at health centers and hospitals, respectively. This is lower than the result of the study conducted in Addis Ababa, which was 26 (60.5 %)^3^ and 14 (73.7 %) of the HCs and 3 (75 % of the hospitals faced stock out of one or more ARV drugs on the day of visited).^18^ The result is somewhat similar to the result of the study conducted by Eyerusaleum; nearly 3/4 of the health facilities faced stock-outs of one or more ARV medicines and test kits on the day of the visit. Another study also shows that all hospitals and HCs faced stock outs of one or more ARV drugs within six months.^30^ The highest percentage of products stocked out at hospitals in the current study may be related to the fact that hospitals have a comparatively larger patient population than health centers and some other problems related to the supplier and at the facilities. Some of the KIs reflected that *“We occasionally experience inconsistent and interrupted supply, which leads to stocking out of some ARV drugs”, inaccurate quantification, delivery of products with short expiration dates, and poor supply systems at central EPSA and its hubs (Informant #2, #4, and #5).*

Pharmaceuticals require good storage conditions that fulfill the standard criteria to maintain product integrity and quality.^9^ This study revealed that 89.25 % and 68.99 % in hospitals and health centers, respectively, met the criteria for acceptable storage conditions. The result is somewhat higher than the result of the study conducted in public health facilities in Addis Ababa, Ethiopia, which indicated only 8 (36.36 %) health facilities met acceptable storage conditions^9^^,^^31^ and the result of the study conducted at public hospitals in Nyamira County, Kenya, showed that lack of proper storage conditions was one of the challenges of inventory management of ART drugs at the surveyed health facilities.^26^ For the case of hospitals, the current result was similar to the result of the study conducted at health facilities in Ethiopia, indicating that the majority of surveyed health facilities had good storage conditions (>80 % of the standard).^12^ However, the poor performance of storage conditions at health centers found in the current study might be due to poor infrastructures; particularly, the lack of adequate storage space was the challenge faced in the management of ARV medicine. KIs indicated that, *“We don't have a separate storage area for expired and damaged ARV medicines. There are no adequate and well-furnished shelves for the storage of available ARV drugs. In such a way, it is difficult to maintain the quality of ARV medicines. Always we asked this issue for higher administrative bodies and offices assigned at facilities, zonal, and regional levels, but still we did not get a solution.” (Informant #3, #4, #8, #9, and #12).*

## 8 Conclusion and Future needs

According to the findings of this study, the availability and utilization of inventory recoding tools and bin cards were satisfied and in-line with the expected targeted value (100 %). However, updating the available bin cards was not satisfied. The study also showed the presence of poor inventory management, i.e., most health facilities had at least one stock out of antiretroviral drugs and at least one emergency order within the last six months prior to data collection. The storage condition of ARV drugs was poor in health centers. However, as compared to health centers, it was good in hospitals (89.52 %). Moreover, as perceived by KIs, this study identified issues such as inadequate infrastructure and administration problems, poor storage conditions (particularly in health centers), a shortage of trained and qualified staff, utilization of a manual reporting system, and a lack of vehicles for drug transportation. Therefore, to improve the performance of ARV drug supply chain management, all the concerned bodies the Ministry of Health, the regional and zonal health bureaus, health facilities, professionals, and researchers should work cooperatively: in maintaining efficient and quality data throughout the ARV drugs supply system, health facilities must be dedicated to updating inventory recording tools, especially when it comes to creating standard storage conditions and providing the necessary space (shelves and pallets) for each facility, as well as recruiting pharmacy professionals, facilitating capacity-building training, and offering ongoing mentorship and supervision. To avoid disruptions in the delivery of ARV medications, the central warehouse should develop plans, maintain adequate inventory at its hub, and effectively collaborate with health care facilities. Supporting the administration of logistics information systems in healthcare facilities requires the introduction of user-friendly tools and automated inventory-controlling systems. Future researchers should focus on the quality and accuracy of data because good decision-making and pharmaceutical supply chain performance are dependent on the availability of quality data.

### 8.1 Strength and limitation of the study

The study used a mixed approach through triangulation of the quantitative data with the qualitative data, which gives better insight for the reader. Future researchers could use this study's information about the current supply chain performance of ARV drugs in the study area as a baseline. On the contrary, due to the nature of the data, this study is limited to descriptive statistics and is not allowed to carry out further inferential statistical analysis and model description for interpreting performance metrics. Besides, due to the insufficient number of previous studies conducted on ARV drug performance of the supply chain in the study area and abroad, it was difficult to compare the results with those of studies conducted in similar settings.

## Funding

Not applicable.

## Consent for publication

Not applicable

## Ethics approval and consent to participate

The study was carried out in accordance with the 1964 Declaration of Helsinki, which was recently revised in October 2013. Before every person answered the questionnaire, their informed consent was requested. Throughout the study, the confidentiality and anonymity of the participants were maintained. Ethical approval was obtained from the research ethics review committee of the College of Medicine and Health Sciences, School of Pharmacy at the University of Gondar with reference number (Ref No: S/A/P/ 41/2023). Then informed consent to participate was obtained from all of the participants working at the selected health facilities.

## CRediT authorship contribution statement

**Meseret Tilahun Zeleke:** Writing – review & editing, Writing – original draft, Visualization, Validation, Methodology, Investigation, Formal analysis, Data curation, Conceptualization. **Berhanemeskel Weldegerima Atsbeha:** Writing – review & editing, Validation, Methodology, Data curation, Conceptualization. **Belachew Yebeyin Melaku:** Writing – review & editing, Methodology, Formal analysis, Data curation, Conceptualization. **Yesuneh Tefera Mekasha:** Writing – review & editing, Visualization, Validation, Data curation. **Abibo Wondie Mekonen:** Writing – review & editing, Writing – original draft, Visualization, Validation, Supervision, Methodology, Formal analysis, Data curation. **Shimelis Dagnachew Nigatu:** Writing – review & editing, Visualization, Supervision.

## Declaration of competing interest

The authors declare no competing interests. All the authors declare that they have no established conflicting financial interests or personal relationships that may have influenced the research presented in this paper.

## Data Availability

Documents supporting this study are available upon request from the corresponding author.

## Appendix A Supplementary data

Supplementary material Image 1
