# Supplementary material for: Performance of antiretroviral drugs supply chain management and related challenges in Amhara National Regional State, Ethiopia. In the case of public health facilities found in the Central Gondar zone
Source: Explor Res Clin Soc Pharm. 2025 Jan 21;17:100570. doi: 10.1016/j.rcsop.2025.100570 (PMC11830304; doi:10.1016/j.rcsop.2025.100570)
Supplement: Supplementary file 1 — Supplementary material [file mmc1.docx]

**Supplementary Table 1**: Percentage of facilities meting specific acceptable storage conditions

| **Storage conditions** | **Response category (%)** | | | |
| --- | --- | --- | --- | --- |
|  | **Health center** | | **Hospital (no)%** | |
|  | **Yes N (%)** | **No N (%)** | **Yes N (%)** | **No N (%)** |
| Pharmaceuticals are arranged according to a logical categorization, e.g. zoning | 21 (91.3%) | 2 (8.7%) | 7 (100%) | 0 |
| Bin Cards are used | 23 (100%) | 0 | 7 (100%) | 0 |
| pharmaceutical separated from unwanted and expired items | 12(52.2%) | 11 (47.8%) | 3( 42.9) | 4 (57.1%) |
| ID labels and expiry dates visible | 21 (91.3%) | 2 (8.7%) | 7 (100%) | 0 |
| Products are arranged in FEFO | 21 (91.3%) | 2 (8.7%) | 7 (100%) | 0 |
| Products are protected from direct sunlight | 18 (78.3%) | 5 (21.7%) | 7 (100%) | 0 |
| The storeroom is maintained in good condition (clean, no trash, strong shelves, and boxes well-organized) | 8 (34.8%) | 15 (65.2%) | 7 (100%) | 0 |
| The current space is sufficient for existing products | 6 (26.1%) | 17 (73.9%) | 5(71.4) | 2(28.6%) |
| Storage area is secured with lock and key | 15 (65.2%) | 8 (34.8%) | 7 (100%) | 0 |
| Storage area is visually free from harmful insects and rodents. | 7 (30.4%) | 16 (69.6%) | 5(71.4) | 2 (28.6%) |
| Products are in good conditions and properly handled | 18 (78.3%) | 5 (21.7%) | 4 (57.1) | 3 ( 42.9) |
| Functional fire safety equipment are available | 16 (69.6%) | 7 (31.4%) | 7 (100%) | 0 |
| Products are stocked at least 20 cm away from the walls and other stacks | 16 (69.6%) | 7 (31.4%) | 7 (100%) | 0 |
| Products are stocked at least 10 cm off the floor | 19 (82.6%) | 4 (17.4 %) | 7 (100%) | 0 |
| Products are stocked with at least 2. 5 m length of the rack | 17 (73.9%) | 6 (26.1%) | 7 (100%) | 0 |
| **Average** | **68.99** | **31.01** | **89.52** | **10.48** |
